# Dopamine signaling negatively regulates striatal phosphorylation of Cdk5 at tyrosine 15 in mice

**DOI:** 10.3389/fncel.2013.00012

**Published:** 2013-02-14

**Authors:** Yukio Yamamura, Ryoma Morigaki, Jiro Kasahara, Hironori Yokoyama, Akie Tanabe, Shinya Okita, Hidetaka Koizumi, Shinji Nagahiro, Ryuji Kaji, Satoshi Goto

**Affiliations:** ^1^Parkinson's Disease and Dystonia Research Center, Tokushima University Hospital, University of TokushimaTokushima, Japan; ^2^Department of Neurobiology and Therapeutics, Graduate School of Pharmaceutical Sciences, Institute of Health Bioscience, University of TokushimaTokushima, Japan; ^3^Department of Neurosurgery, Graduate School of Medical Sciences, Institute of Health Biosciences, University of TokushimaTokushima, Japan; ^4^Department of Clinical Neuroscience, Graduate School of Medical Sciences, Institute of Health Biosciences, University of TokushimaTokushima, Japan

**Keywords:** cyclin-dependent kinase 5, phosphorylation, striatum, cell signaling

## Abstract

Striatal functions depend on the activity balance between the dopamine and glutamate neurotransmissions. Glutamate inputs activate cyclin-dependent kinase 5 (Cdk5), which inhibits postsynaptic dopamine signaling by phosphorylating DARPP-32 (dopamine- and cAMP-regulated phosphoprotein, 32 kDa) at Thr75 in the striatum. c-Abelson tyrosine kinase (c-Abl) is known to phosphorylate Cdk5 at Tyr15 (Tyr15-Cdk5) and thereby facilitates the Cdk5 activity. We here report that Cdk5 with Tyr15 phosphorylation (Cdk5-pTyr15) is enriched in the mouse striatum, where dopaminergic stimulation inhibited phosphorylation of Tyr15-Cdk5 by acting through the D2 class dopamine receptors. Moreover, in the 1-methyl-4-phenyl-1,2,4,6-tetrahydropyridine (MPTP) mouse model, dopamine deficiency caused increased phosphorylation of both Tyr15-Cdk5 and Thr75-DARPP-32 in the striatum, which could be attenuated by administration of L-3,4-dihydroxyphenylalanine and imatinib (STI-571), a selective c-Abl inhibitor. Our results suggest a functional link of Cdk5-pTyr15 with postsynaptic dopamine and glutamate signals through the c-Abl kinase activity in the striatum.

## Introduction

Cyclin-dependent kinase 5 (Cdk5) is a member of the Cdk family of serine/threonine kinases and is abundantly expressed in the brain (Dhavan and Tsai, 2001). Besides the essential role of Cdk5 in neuronal positioning and synaptogenesis during brain development, Cdk5 has also been involved in the cell signaling and survival in adult brains (Dhavan and Tsai, 2001; Smith and Tsai, 2002; Dhariwala and Rajadhyaksha, 2008; Hisanaga and Endo, 2010). In the striatum, Cdk5 plays a regulatory role in the dopamine and glutamate transmissions that are integrated by DARPP-32 (dopamine- and cAMP-regulated phosphoprotein, 32 kDa) (Greengard, 2001). Glutamate inputs activate Cdk5, which inhibits postsynaptic dopamine D1 receptor (D1R)-mediated signaling by phosphorylating DARPP-32 at Thr75 (Thr75-DARPP-32) in the striatum, since DARPP-32 with Thr75 phosphorylation (DARPP-32-pThr75) functions as an inhibitor of cAMP-dependent protein kinase A (PKA). Dysregulation of Cdk5 activity has been implicated in striatal dopamine disorders that include Parkinson's disease (PD) (Chergui et al., 2004) and drug addiction (Bibb et al., 2001; Takahashi et al., 2005); however, it is not fully understood how Cdk5 interacts with dopamine signaling in the striatum.

One of the regulatory mechanisms that modulate the Cdk5 kinase activity is phosphorylation of conserved residues (Dhavan and Tsai, 2001). c-Abelson kinase (c-Abl), a non-receptor tyrosine kinase related to the Src family, is the known kinase that phosphorylates Cdk5 at Tyr15 (Tyr15-Cdk5) and thereby increases its kinase activity (Zukerberg et al., 2000; Dhavan and Tsai, 2001; Zhang et al., 2007). Impaired activity of c-Abl participates in the pathogenesis of several neurodegenerative diseases (Schlatterer et al., 2011). We recently found that Cdk5 with phosphorylation of Tyr15 (Cdk5-pTyr15) is enriched in the striatum (Morigaki et al., 2011). This strategic localization of Cdk5-pTyr15 suggests a possible role of the c-Abl/Cdk5 signaling in modulation of postsynaptic dopamine and glutamate transmissions in the striatum.

Here, we show that in the mouse striatum, dopamine receptor stimulation negatively regulates phosphorylation of Tyr15-Cdk5 most likely through a D2R-mediated mechanism. We also provide an evidence that in the 1-methyl-4-phenyl-1,2,4,6-tetrahydropyridine (MPTP) mouse model with striatal dopamine deficiency, a c-Abl kinase inhibitor imatinib (STI-571) (Capdeville et al., 2002) attenuates abnormally increased striatal phosphorylation of both Tyr15-Cdk5 and Thr75-DARPP-32, as does L-3,4-dihydroxyphenylalanine (L-dopa). These findings have important implications for understanding the mechanisms by which the Cdk5/DAPP-32-Thr75 signaling regulates the striatal functions.

## Materials and methods

### Experimental animals

All procedures involving experimental mice were approved by the Ethical Review Committee of the University of Tokushima. Male C57BL/6 mice aged at 8–9 weeks (Nihon SLC Co., Shizuoka, Japan) were used. Mice were housed under a 12 h-light and 12 h-dark cycle with access to food and tap water *ad libitum*. The total number of mice used in this study was 350.

### MPTP administration

Mice were injected intraperitoneally 4 times in one day with MPTP hydrochloride (20 mg/kg of free base; Sigma–Aldrich, St Louis, MO) at 2 h intervals (Yokoyama et al., 2010). Saline-treated mice received an equivalent volume of 0.9% saline. Our previous study showed that maximal neurodegenerative effects of MPTP on the nigral dopaminergic cells were observed at the 3 days time-point after administration of MPTP (Aoki et al., 2009).

### L-dopa administration

Mice received single intraperitoneal injections of L-dopa hydrochloride (15 mg/kg of free base; Sigma-Aldrich) dissolved in 0.9% saline containing 0.5% carboxymethyl cellulose 3 days after administration of MPTP or saline. The used dose of L-dopa was chosen based on the findings reported previously (Chartoff et al., 2001). Vehicle-treated mice received an equivalent volume of 0.9% saline containing 0.5% carboxymethyl cellulose. They were pre-treated with single intraperitoneal injections of benserazide (12.5 mg/kg; Sigma–Aldrich) dissolved in 0.9% saline 20 min before administration of L-dopa or vehicle.

### Imatinib administration

Mice received single intraperitoneal injections of imatinib mesylate (25 mg/kg; LKT Laboratories, St. Paul, MN) dissolved in 0.9% saline containing 10% dimethylsulfoxide 3 days after administration of MPTP or saline. Vehicle-treated mice received an equivalent volume of 0.9% saline containing 10% dimethylsulfoxide.

### Western blot analysis

Mice were sacrificed by cervical dislocation 30 min after intraperitoneal administration of apomorphine hydrochloride, (5 or 10 mg/kg of free base; Sigma–Aldrich), A-68930 hydrochloride (2 mg/kg of free base; Sigma–Aldrich), SCH-23390 hydrochloride (0.5 mg/kg of free base; Sigma–Aldrich), quinpirole hydrochloride (5 mg/kg of free base; Sigma–Aldrich), raclopride tartrate (1 mg/kg of free base; Sigma–Aldrich), L-dopa (15 mg/kg) with benserazide (12.5 mg/kg), or imatinib mesylate (25 mg/kg or 10 mg/kg). Western blot analysis was carried out according to the method described previously (Kasahara et al., 2001). Briefly, striatal tissue samples from deeply anesthetized mice were homogenized in 50 mM Tris-HCl buffer, pH 7.5, containing 0.5 M NaCl, 0.5% Triton X-100, 10 mM EDTA, 4 mM EGTA, 1 mM Na_3_VO_4_, 30 mM Na_4_P_2_O_7_, 50 mM NaF, 0.1 mM leupeptin, 0.075 mM pepstatin A, 0.05 mg/ml trypsin inhibitor, 1 mM phenylmethanesulfonyl fluoride, 100 nM calyculin A, and 1 mM dithiothreitol. After centrifugation at 21,500 × *g* for 10 min, the protein lysates were resuspended in 100 mM NaH_2_PO_4_, pH 6.0, I mM EDTA, 1% 2-mercaptoethanol, 0.1% sodium dodecyl sulfate, to 1 mg/ml final protein concentration and was heated at 99°C for 3 min. Specific antibodies against Cdk5-pTyr15 (1:1000; Abcam, Cambridge, UK), Cdk5 (1:1000; Santa Cruz Biotechnology, Santa Cruz, CA), DARPP-32-pThr75 (1:1000; Cell Signaling, Danvers, MA), DARPP-32-pThr34 (1:1000; Cell Signaling), DARPP-32 (1:1000; Cell Signaling) were used. The monospecificity of these antibodies was confirmed in our previous reports (Sako et al., 2010; Morigaki et al., 2011). Anti-β-actin antibody (1:1000; Sigma–Aldrich) was used to adjust equal amounts of protein loading into each well. Gel images were captured using a lumino-imaging analyzer LAS-4000 (Fuji, Tokyo, Japan). Optical densities were determined using a computerized image analysis system (Dolphin-DOC; Kurabo, Osaka, Japan) (Yokoyama et al., 2010).

### Tissue preparation and immunostaining

Mice were injected intraperitoneally with a lethal dose of pentobarbital (Sigma, St Louis, MO) 30 min after drug administration. They were then transcardially perfused with 0.01 M phosphate-buffered saline (PBS) at pH 7.4, followed by cold 4% paraformaldehyde in 0.1 M phosphate buffer (PB) at pH 7.4. The brains were removed, post-fixed overnight in the same fixative at 4°C, and stored in a 10–30% sucrose gradient in 0.1 M PB at 4°C for cryoprotection. Sections were cut on a cryostat at 15 μm-thickness, and stored in PBS containing 0.05% NaN_3_ until use. Immunofluorescence staining was carried out with free-floating brain sections (Morigaki et al., 2011). Primary antibodies against Cdk5-pTyr15 (1:20,000; Santa Cruz), tyrosine hydroxylase (TH, 1:100,000) (Sato et al., 2008), and DARPP-32-Thr75 (1:20,000; Cell Signaling) (Sako et al., 2010) were used. The bound primary antibodies were detected using the Histofine Simple Stain Kit (Nichirei, Tokyo, Japan) and Tyramide Signal Amplification (TSA) system with Cyanine3 or Fluorescein (Perkin Elmer, Shelton, CT) (Morigaki et al., 2011).

For double immunofluorescence staining Cdk5-pTyr15 and μ-opioid receptor (MOR), dual antigen detection with the TSA system was carried out according to the method that we reported previously (Okita et al., 2012). Briefly, sections were first incubated in PBS containing 3% BSA and anti-Cdk5-pTyr15 antibodies (1:20,000; Santa Cruz). The bound primary antibodies were detected by the Histofine Simple Stain Kit (Nichirei, Tokyo, Japan) and the TSA system with Cyanine3 (Perkin Elmer). To remove the bound antibodies, the stained sections were then incubated in 0.1 M glycine-HCl (pH 2.2) at room temperature for 30 min. After incubation in PBS for 1 h, the sections were incubated in PBS containing 3% BSA and antibodies for MOR (1:100,000; Millipore, St. Louis, MO, USA). The bound antibodies were detected by the Histofine Simple Stain Kit (Nichirei) and the TSA system with Fluorescein (Perkin Elmer).

### Digital imaging and densitometry

Digital microscopic images were captured using an Olympus BX51 microscope (Olympus, Tokyo, Japan), imported into Adobe Photoshop CS4, and processed digitally. The optical densities of immunoreactive products were measured as gray levels on non-colored digital images (Sato et al., 2008; Morigaki et al., 2011). For each animal (*n* = 5), density measurement was made in a striatal section at the level of 0.9–1.1 mm anterior to bregma, according to the atlas of Hof et al. (2000).

### Statistical analysis

All experimental values were expressed as means ± S.E.M. Statistical significance was evaluated by two-tailed Student's *t*-test, or One-Way ANOVA with Bonferroni–Dunn or Fisher's PLSD *post hoc* test. The significance level was set at *P* < 0.05.

## Results

### Enrichment of Cdk5-pTyr15 in the striatum

**Figure 1:**
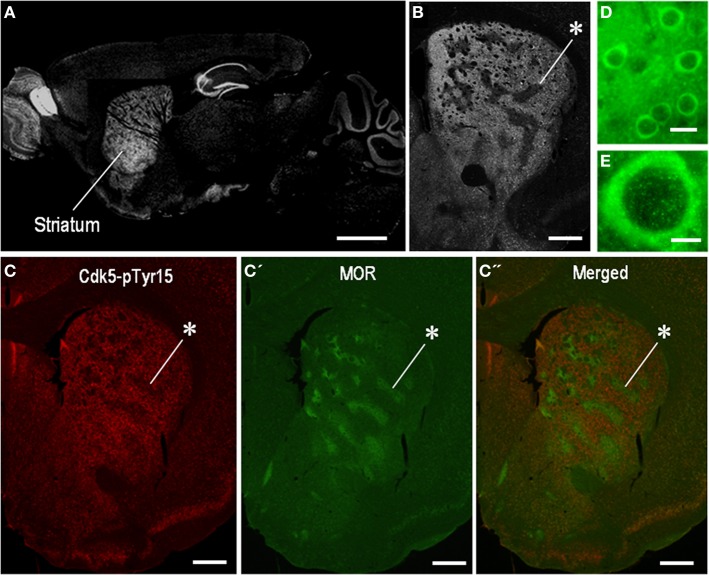
**Localization of Cdk5-p Tyr15 in the mouse striatum. (A)** Photomontage of parasagittal brain sections stained for Cdk5-pTyr15. **(B)** Photomicrograph of a striatal section stained for Cdk5-pTyr15. Note that the matrix compartment is enriched in Cdk5-pTyr15 as compared to the striosomes. The asterisk indicates an example of striosomes poor in Cdk5-pTyr15 labeling. **(C–C”)** Double immunofluorescence staining for Cdk5-pTyr15 **(C)** and MOR **(C')**, and merged **(C”)**. A corresponding striosome is indicated by the asterisks. **(D** and **E)** Photomicrographs of striatal neurons immunoreactive for Cdk5-pTyr15. Cdk5-pTyr15 labeling is found in neuronal soma, processes, and nuclei of striatal neurons. Scale bars: **(A)**, 1 mm; **(B)** and **(C–C”)**, 500 μm; **(D)**, 20 μm; **(E)**, 5 μm.

We first determined the localization pattern of Cdk5-pTyr15 in the brain of mice used in this study. Among brain regions, strong Cdk5-pTyr15 labeling was found in the striatum that consists of the dorsal striatum (caudoputamen), nucleus accumbens and olfactory tubercle (Figure 1A). In the dorsal striatum, Cdk5-pTyr15 immunostaining exhibited an inhomogeneous distribution with heightened labeling in the matrix compartment relative to the striosomes (Figures 1B,C). At the cellular level (Figures 1D,E), Cdk5-pTyr15 immunoreactivity was found in neuronal soma, processes, and nuclei of striatal neurons. Thus, Cdk5-pTyr15 appears as a striatal-enriched phosphoprotein, as does DARPP-32 (Ouimet et al., 1998).

### Dopamine receptor stimulation inhibits striatal phosphorylation of Cdk5 at Tyr15

**Figure 2:**
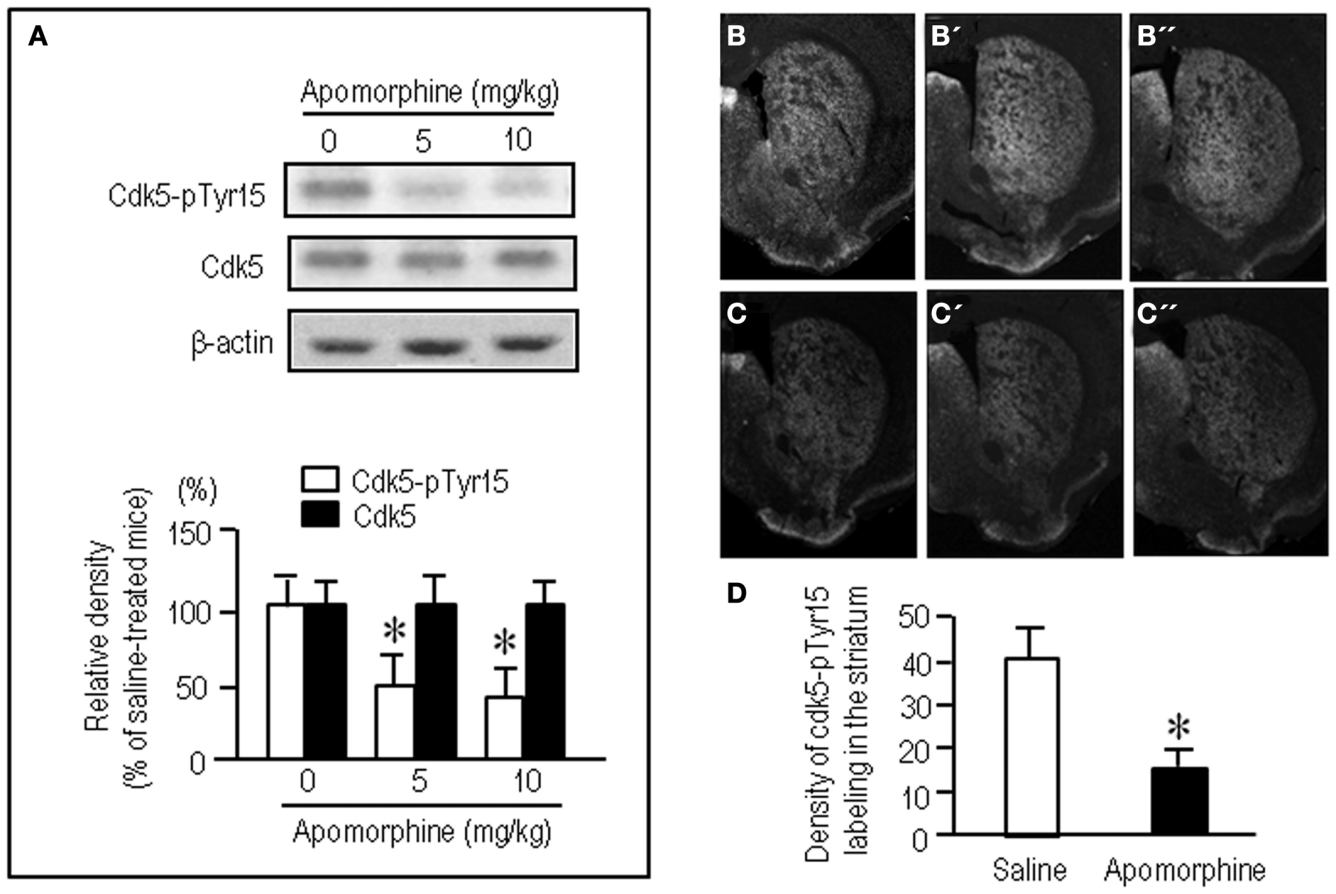
**Dopamine receptor stimulation inhibits striatal phosphorylation of Cdk5 at Tyr15. (A)** Western blot analysis on the effects of apomorphine on striatal levels of Cdk5-pTyr15. Mice received saline or the indicated amounts of apomorphine 30 min before sacrifice. Values are means ± S.E.M. (*n* = 5). ^*^P < 0.005 versus saline-treated mice; two tailed Student's *t*-test. **(B–D)** Immunohistochemical assessment of the effects of apomorphine on striatal density of Cdk5-pTyr15 labeling. **(B–B”)** Frontal sections stained for Cdk5-pTyr15 in the anterior (**B**; 1.3 mm anterior to bregma), middle (**B'**; 1.0 mm anterior to bregma), and posterior (**B”**; 0.1 mm posterior to bregma) levels of the striatum from a saline-treated mouse. **(C–C”)** Frontal sections stained for Cdk5-pTyr15 in the rostral (**C**; 1.3 mm anterior to bregma), middle (**C'**; 1.0 mm anterior to bregma), and caudal (**C”**; 0.1 mm posterior to bregma) levels of the striatum from an apomorphine-treated mouse. **(D)** Optical density measurements of striatal Cdk5-pTyr15 labeling in saline- and apomorphine-treated mice. Values are means ± S.E.M. (*n* = 5). ^*^P < 0.005 versus saline-treated mice; two-tailed Student's *t*-test.

**Figure 3:**
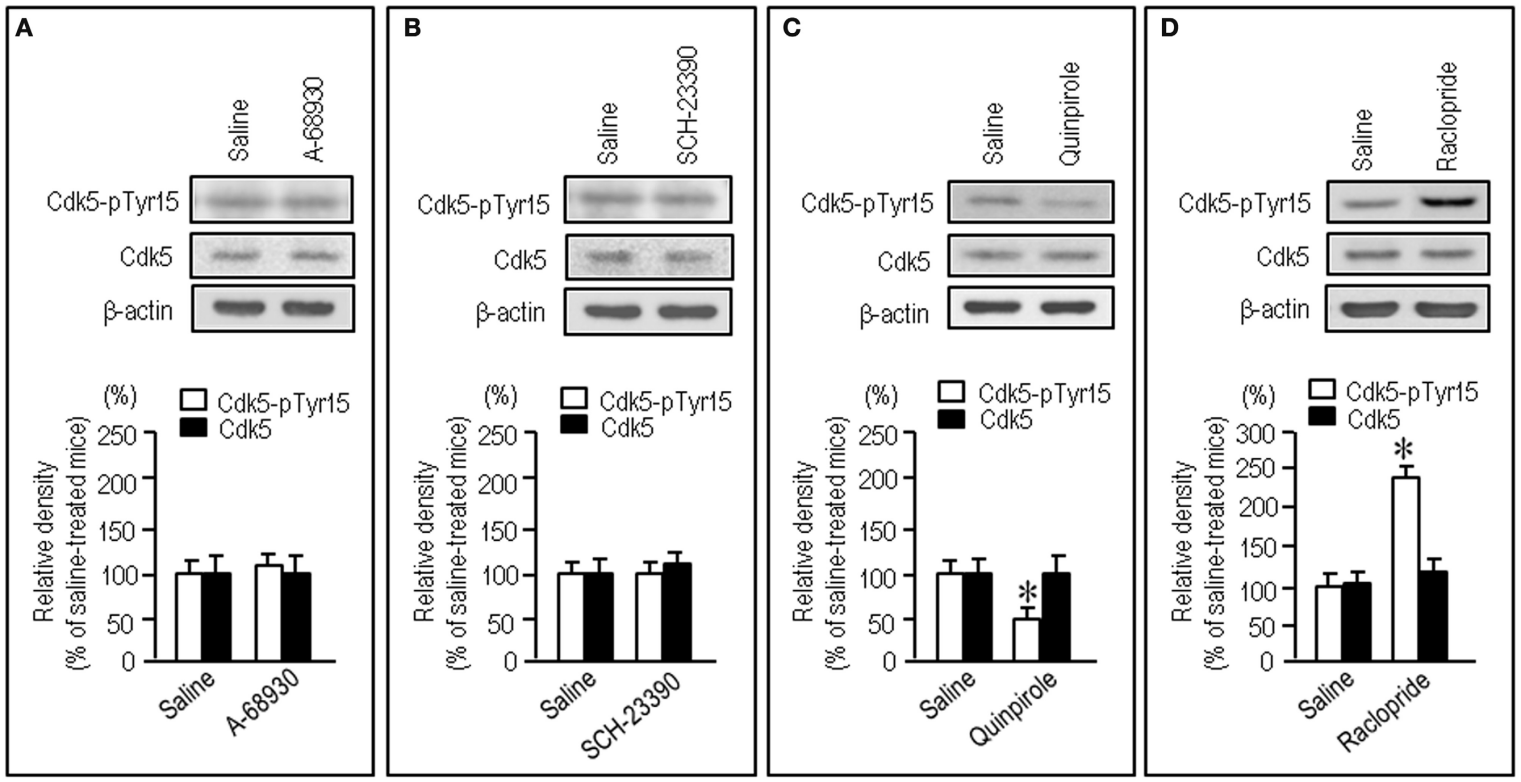
**Negative regulation of striatal phosphorylation of Tyr15-Cdk5 through a D2R-mediated mechanism.** Western blot analysis was carried out on the striatal extracts from mice that received saline, A-68930 (2 mg/kg), SCH-23390 (0.5 mg/kg), quinpirole (5 mg/kg), or raclopride (1 mg/kg), 30 min before sacrifice. Effects of administration of A-68930 **(A)**, SCH-23390 **(B)**, quinpirole **(C)**, or raclopride **(D),** on striatal levels of Cdk5-pTyr15 are shown. Values are means ± S.E.M. (*n* = 5). ^*^P < 0.005 versus saline-treated mice; two tailed Student's *t*-test.

To test whether striatal phosphorylation of Tyr15-Cdk5 was altered by dopaminergic stimulation, we performed the western-blot assay for Cdk5-pTyr15 expression after treatment with apomorphine, an agonist for both the D1Rs and D2Rs. A significant reduction of the striatal levels of Cdk5-pTyr15 was found following administration of apomorphine at a dose of 5 mg/kg or 10 mg/kg (Figure 2A; *P* < 0.005, Student's *t*-test). Immunohistochemical study on frontal sections from anterior to posterior of the forebrain also showed a decreased density of Cdk5-pTyr15 staining in the striatum of mice that received apomorphine (10 mg/kg) (Figures 2B,C). Densitometric measurements confirmed this (Figure 2D; *P* < 0.005, Student's *t*-test). We next examined whether dopaminergic stimulation could cause reduced expression of Cdk5-pTyr15 by acting on the D1 or D2 class receptors (Figure 3). Western blot analysis revealed no apparent changes in the striatal levels of Cdk5-pTyr15 following administration of a D1 receptor agonist A-68930 (2 mg/kg) (Figure 3A; *P* > 0.05, Student's *t*-test), or a D1 receptor antagonist SCH-23390 (0.5 mg/ml) (Figure 3B; *P* > 0.05, Student's *t*-test). By contrast, striatal levels of Cdk5-pTyr15 were significantly decreased following administration of a D2 receptor agonist quinpirole (5 mg/kg) (Figure 3C; *P* < 0.005, Student's *t*-test), and they were significantly increased following administration of a D2 receptor antagonist raclopride (1 mg/kg) (Figure 3D; *P* < 0.005, Student's *t*-test). These findings suggest that dopamine signal can inhibit striatal phosphorylation of Tyr15-Cdk5 and this process most likely occurs through a D2R-mediated mechanism.

### L-dopa effects on striatal phosphorylation of Cdk5 and DARPP-32 in MPTP mice

**Figure 4:**
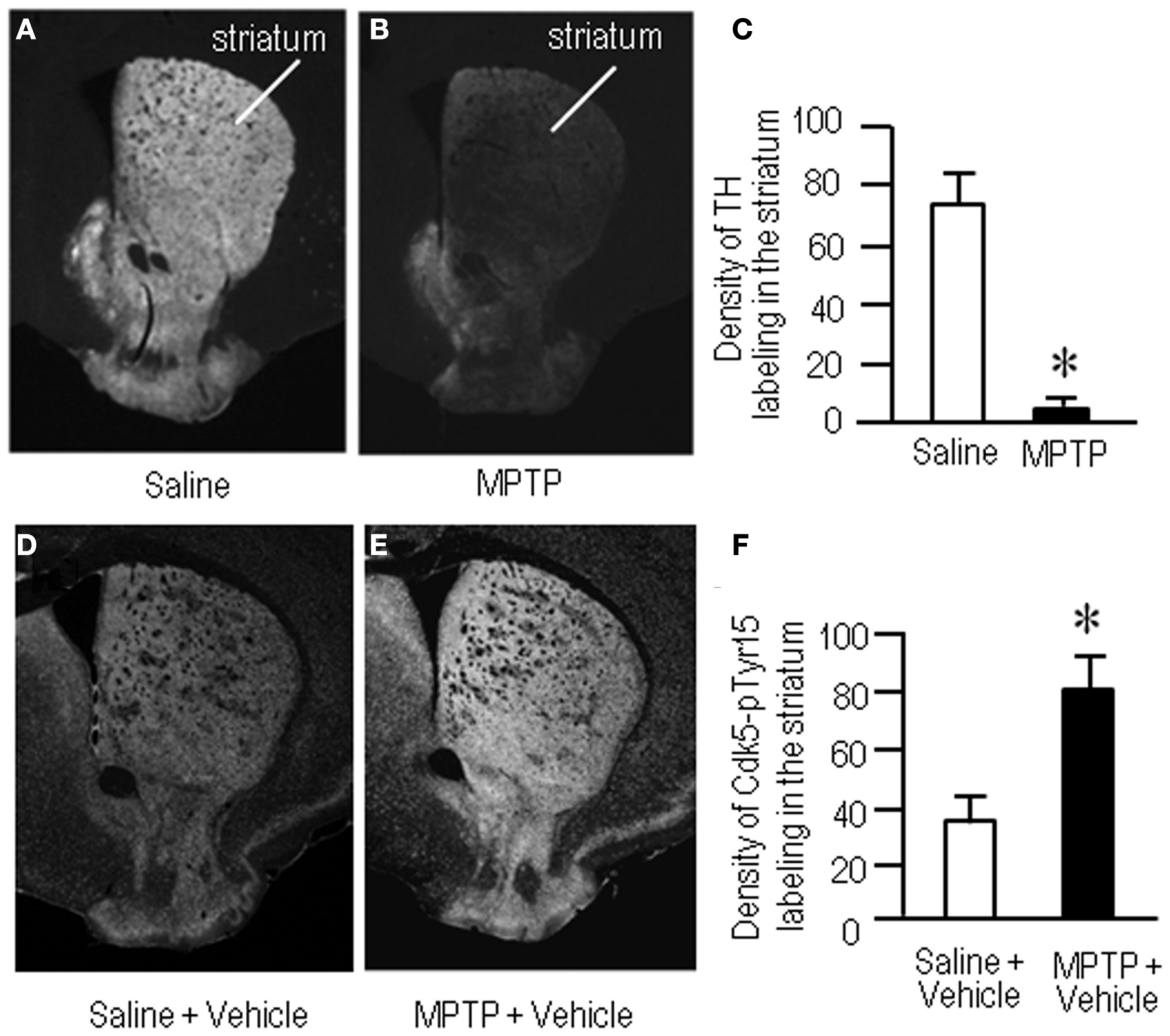
**Loss of dopaminergic inputs and increased phosphorylation of Tyr15-Cdk5 in the striatum in MPTP mice.** Striatal sections were prepared and subjected to the immunohistochemical study (see “Materials and Methods”). **(A–C)** Severe loss of TH-immunoreactive afferents in the striatum in MPTP mice. Representative images of striatal sections (0.0–1.0 mm anterior to bregma) immunostained for TH from saline- **(A)** and MPTP-treated **(B)** mice. **(C)** Optical density measurements of striatal TH labeling in saline- and MPTP-treated mice. Values are means ± S.E.M. (*n* = 5). ^*^P < 0.001 versus saline-treated mice, two tailed Student's *t*-test. **(D–F)** Increased density of striatal Cdk5-pTyr15 labeling in MPTP mice. Representative images of striatal sections (0.0–1.0 mm anterior to bregma) immunostained for Cdk5-pTyr15 from saline- **(D)** and MPTP-treated **(E)** mice. **(F)** Optical density measurements of striatal Cdk5-pTyr15 labeling in saline- and MPTP-treated mice. Values are mean ± S.E.M. (*n* = 5). ^*^P < 0.01 versus saline-treated mice, two tailed Student's *t*-test.

**Figure 5:**
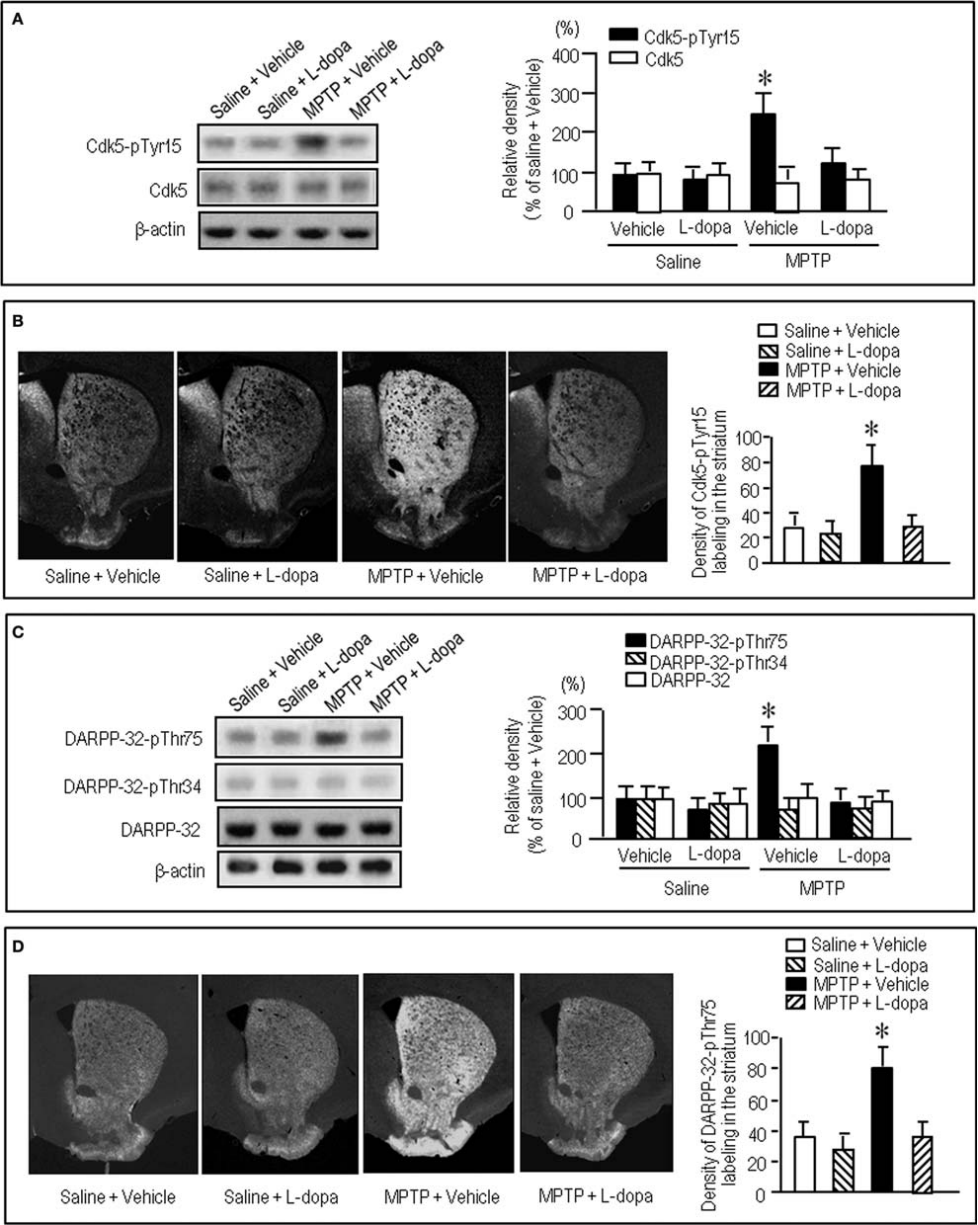
**Effects of L-dopa on striatal phosphorylation of Cdk5 and DARPP-32 in MPTP mice.** Saline- or MPTP-treated mice received vehicle or L-dopa 30 min before sacrifice. Striatal tissue extracts and sections were prepared and subjected to western blotting and immunohistochemical staining (see “Materials and Methods”). **(A)** Western blot analysis of striatal levels of Cdk5-pTyr15 and Cdk5 in mice treated with saline + vehicle, saline + L-dopa, MPTP + vehicle, or MPTP + L-dopa. Values are means ± S.E.M. (*n* = 5–6). ^*^P < 0.001 versus mice treated with saline + vehicle, saline + L-dopa, or MPTP + L-dopa; One-Way ANOVA [*F*_(3.18)_ = 31.06] followed by Bonferroni–Dunn test. **(B)** Immunohistochemical study on striatal labeling for Cdk5-pTyr15 in mice treated with saline + vehicle, saline + L-dopa, MPTP + vehicle, or MPTP + L-dopa. Representative images of the striatal sections (0.0–1.0 mm anterior to bregma) stained for Cdk5-pTyr15 from each group are shown. Densitometric analysis was made on a striatal section from each mouse in a group of saline + vehicle, saline + L-dopa, MPTP + vehicle or MPTP + L-dopa. Values are means ± S.E.M. (*n* = 5). ^*^P < 0.005 versus mice treated with saline + vehicle, saline + L-dopa, or MPTP + L-dopa; One-Way ANOVA [*F*_(3.16)_ = 26.84] followed by Fisher's PLSD test. **(C)** Western blot analysis of striatal levels of DARPP-32-pThr75, DARPP-32-pThr34, and DARPP-32 in mice treated with saline + vehicle, saline + L-dopa, MPTP + vehicle, or MPTP + L-dopa. Values are means ± S.E.M. (*n* = 4–7). ^*^P < 0.01 versus mice treated with saline + vehicle, saline + L-dopa, or MPTP + L-dopa; One-Way ANOVA [*F*_(3.18)_ = 6.96] followed by Bonferroni–Dunn test. **(D)** Immunohistochemical study on striatal labeling for DARPP-32-pThr75 in mice treated with saline + vehicle (*n* = 5), saline + L-dopa (*n* = 5), MPTP + vehicle (*n* = 5), or MPTP + L-dopa (*n* = 5). Representative images of the striatal sections (0.0–1.0 mm anterior to bregma) stained for DARPP-32-pThr75 from each group are shown. Densitometric analyses were made on a striatal section from each mouse in a group of saline + vehicle, saline + L-dopa, MPTP + vehicle or MPTP + L-dopa. Values are means ± S.E.M (*n* = 5). ^*^P < 0.005 versus mice treated with saline + vehicle, saline + L-dopa, or MPTP + L-dopa; One-Way ANOVA [*F*_(3.16)_ = 19.62] followed by Fisher's PLSD test.

To gain further insight into the interactions between the c-Abl/Cdk5 signaling and the dopamine and glutamate signal cascades, we conducted an experiment in MPTP mice. Immunohistochemical study revealed severe loss of dopaminergic afferents labeled for TH in the striatum of the MPTP mice (Figures 4A–C). By contrast, an increased density of Cdk5-pTyr15 labeling in the striatum was found in MPTP mice when compared with control mice (Figures 4D–F; *P* < 0.01, Student's *t*-test). It was evident in the entire striatum that includes dorsal striatum, nucleus accumbens and olfactory tubercle. Western blot analysis also revealed a significant increase in the striatal levels of Cdk5-pTyr15 (Figure 5A; *P* < 0.005, ANOVA), but not Cdk5 (Figure 5A; *P* > 0.05, ANOVA), in MPTP mice as compared to saline-treated mice. It was also noted that in MPTP mice L-dopa treatment significantly attenuated the abnormally elevated striatal levels of Cdk5-pTyr15 (Figure 5A; *P* < 0.005, ANOVA). This antagonistic action of L-dopa on striatal phosphorylation of Tyr15-Cdk5 in MPTP mice was also found in the immunohistochemical assessment (Figure 5B).

We next examined on striatal phosphorylation of DARPP-32 in MPTP mice (Figures 5C,D). Western blot analysis revealed a significant increase in striatal phosphorylation of DARPP-32 at Thr75, the substrate site targeted by Cdk5, in MPTP mice, as compared to saline-treated mice (Figure 5C; *P* < 0.05, ANOVA). No apparent difference in striatal levels of DARPP-32-pThr34 (Figure 5C; *P* > 0.05, ANOVA) and DARPP-32 (Figure 5C; *P* > 0.05, ANOVA) was found between the MPTP mice and the saline-treated mice. L-Dopa treatment significantly reduced the abnormally elevated striatal levels of DARPP-32-pThr75 in MPTP mice (Figure 5C; *P* < 0.05, ANOVA). Immunohistochemical analysis also documented that L-dopa reversed increased density of striatal labeling for DARPP-32-pThr75 in MPTP mice (Figure 5D).

### Imatinib effects on striatal phosphorylation of Tyr15-Cdk5 and Thr75-DARPP-32 in MPTP mice

**Figure 6:**
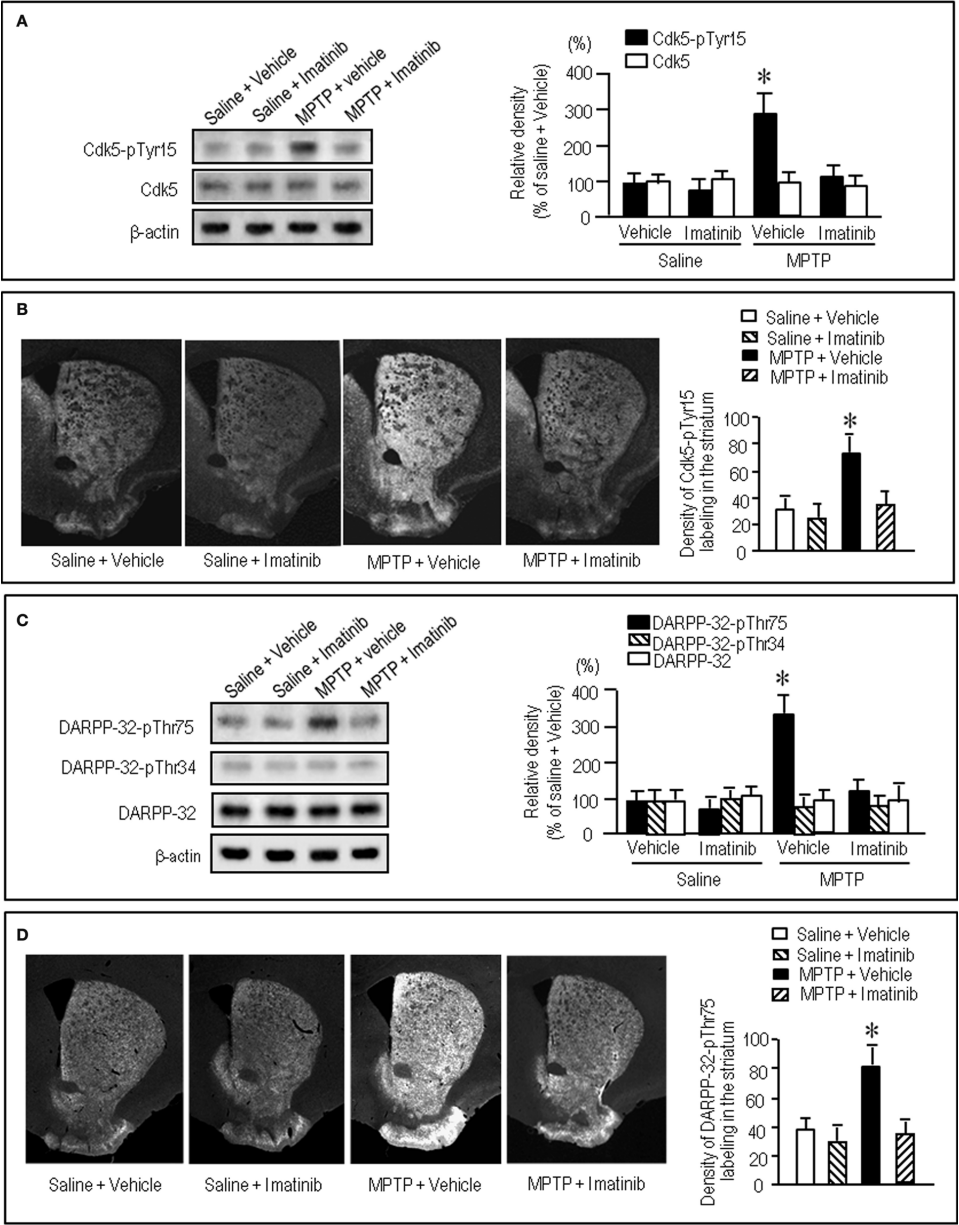
**Effects of imatinib on striatal phosphorylation of Cdk5 and DARPP-32 in MPTP mice.** Saline- or MPTP-treated received vehicle or imatinib 30 min before sacrifice. Striatal tissue extracts and sections were prepared and subjected to western blotting and immunohistochemical staining (see “Materials and Methods”). **(A)** Western blot analysis of striatal levels of Cdk5-pTyr15 and Cdk5 in mice treated with saline + vehicle, saline + imatinib, MPTP + vehicle, or MPTP + imatinib. Values are mean ± S.E.M. (*n* = 4–5). ^*^P < 0.05 versus mice treated with saline + vehicle, saline + imatinib, or MPTP + imatinib; One-Way ANOVA [*F*_(3.14)_ = 9.28] followed by Bonferroni–Dunn test. **(B)** Immunohistochemical study on striatal labeling for Cdk5-pTyr15 in mice treated with saline + vehicle, saline + imatinib, MPTP + vehicle or MPTP + imatinib. Representative images of the striatal sections (0.0–1.0 mm anterior to bregma) stained for Cdk5-pTyr15 from each group are shown. Densitometric analyses were made on a striatal section from each mouse in a group of saline + vehicle, saline + imatinib, MPTP + vehicle or MPTP + imatinib. Values are means ± S.E.M. (*n* = 5). ^*^P < 0.01 versus mice treated with saline + vehicle, saline + imatinib, or MPTP + imatinib; One-Way ANOVA [*F*_(3.16)_ = 9.82] followed by Fisher's PLSD test. **(C)** Western blot analysis of striatal levels of DARPP-32-pThr75, DARPP-32-pThr34, and total DARPP-32 in mice treated with saline + vehicle, saline + imatinib, MPTP + vehicle, or MPTP + imatinib. Values are means ± S.E.M. (*n* = 4–5). ^*^P < 0.05 versus mice treated with saline + vehicle, saline + imatinib, or MPTP + imatinib; One-Way ANOVA [*F*_(3.13)_ = 4.13] followed by Bonferroni–Dunn test. **(D)** Immunohistochemical study on striatal labeling for DARPP-32-pThr75 in mice treated with saline + vehicle, saline + imatinib, MPTP + vehicle or MPTP + imatinib. Representative images of the striatal sections (0.0–1.0 mm anterior to bregma) stained for DARPP-32-pThr75 from each group are shown. Densitometric analyses were made on a striatal section from each mouse in a group of saline + vehicle, saline + imatinib, MPTP + vehicle or MPTP + imatinib. Values are means ± S.E.M. (*n* = 5). ^*^P < 0.05 versus mice treated with saline + vehicle, saline + imatinib, or MPTP + imatinib; One-Way ANOVA [*F*_(3.16)_ = 5.12] followed by Fisher's PLSD test.

We next asked if a selective c-Abl inhibitor imatinib (STI-571) could modulate striatal phosphorylation of Cdk5 and DARPP-32. Western blot analysis revealed that in MPTP mice abnormally elevated striatal levels of Cdk5-pTyr15 was significantly attenuated by administration of imatinib at a dose of 25 mg/kg (Figure 6A; *P* < 0.005, ANOVA), but not 10 mg/kg (data not shown). This was consistent with the results obtained with immunohistochemistry (Figure 6B). Imatinib treatment also significantly attenuated abnormally increased striatal levels of DARPP-32-pThr75 in MPTP mice, as determined by western blot (Figure 6C; *P* < 0.05, ANOVA) and immunohistochemical (Figure 6D; *P* < 0.05, ANOVA) analyses.

Taken together, dopamine deficiency caused an increased phosphorylation of both Tyr15-Cdk5 and Thr75-DARPP-32 in the striatum, which could be ameliorated by administration of L-dopa and imatinib.

## Discussion

**Figure 7:**
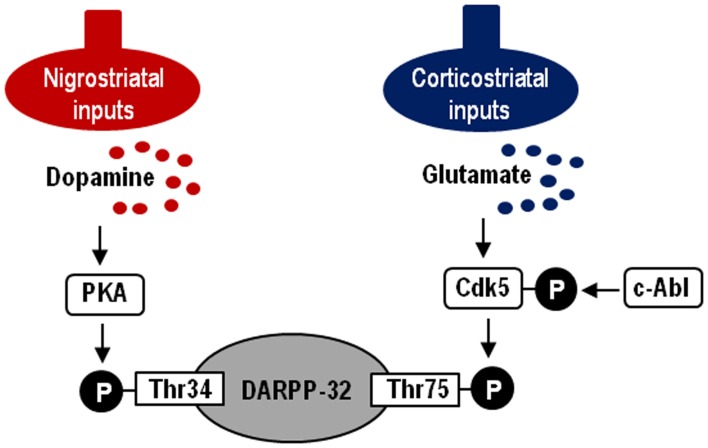
**Hypothesized model of striatal Cdk5/DARPP-32 signal regulations.** Depicted are postsynaptic dopamine/PKA/Thr34-DARPP-32 and glutamate/Cdk5/Thr75-DARPP-32 signaling cascades. c-Abl is the known kinase to phosphorylate Cdk5 at Tyr15.

In this study, we demonstrate a functional link of Cdk5-pTyr15, a striatal-enriched phosphoprotein, with postsynaptic dopamine and glutamate signal cascades in the striatum. Striatal functions depend on an activity balance between dopamine and glutamate transmissions that produce opposing physiological effects (Greengard, 2001; Chergui et al., 2004) (for reference see Figure 7). DARPP-32 integrates the activities of dopaminergic and glutamatergic transmissions (Svenningsson et al., 2004; Fernandez et al., 2006), and it is therefore thought to play as a key regulator for striatal activities (Bonito-Oliva et al., 2011). This striatal-enriched protein can function as either a protein phosphatase inhibitor or a kinase inhibitor, depending on whether Thr34-DARPP-32 or Thr75-DARPP-32 is phosphorylated (Greengard, 2001; Yger and Girault, 2011). In this scenario, glutamate inputs are thought to exert an antagonistic action on postsynaptic dopamine signaling by activating Cdk5 to phosphorylate Thr75-DARPP-32 in the striatum. DARPP-32-pThr75 functions as an inhibitor of PKA that is a key mediator of the D1R-signals. We here showed that the c-Abl inhibitor imatinib attenuated abnormally elevated striatal levels of Cdk5-pTyr15 and DARPP-32-pThr75 in MPTP mice, as did L-dopa. As c-Abl is the known kinase that phosphorylate Tyr15-Cdk5 (Zukerberg et al., 2000; Dhavan and Tsai, 2001; Zhang et al., 2007), these observations suggest an involvement of the c-Abl/Cdk5-pTyr15 signaling in the mechanism by which glutamate inputs activate Cdk5 to increase phosphorylation of Thr75-DARPP-32. Our present data also indicate that dopamine receptor stimulation negatively regulates the glutamate/Cdk5 cascade by inhibiting striatal phosphorylation of Tyr15-Cdk5; this process most likely occurs through a D2R-mediated mechanism. This might be in accordance with the reported findings that by acting through the D2Rs, dopamine signaling reduces presynaptic glutamate release and it also attenuates postsynaptic cellular responsiveness to glutamate receptor stimulation in the striatum (Surmeier et al., 2007). However, it is currently unknown how glutamate inputs activate c-Abl to increase striatal phosphorylation of Cdk5 on Tyr15. Identification of the molecular basis for the glutamate-mediated activation of c-Abl kinase in the striatum might remain an intriguing possibility.

Among striatal disorders, PD is a representative movement disorder that results from striatal dopamine deficiency. In the striatum under resting conditions, Thr75-DARPP-32 is highly phosphorylated, whereas Thr34-DARPP-32 is only slightly phosphorylated (Greengard, 2001; Sako et al., 2010). This suggests that tonic activity of the glutamate/Cdk5 pathway might be responsible for maintaining Thr75-DARPP-32 in a phosphorylated state, thereby inhibiting the D1R-PKA signaling in the striatum (Greengard, 2001). In addition, landmark reports showed that in a rodent model of PD, striatal dopamine deficiency had no effect on phosphorylation of Thr34-DARPP-32, but significantly increased that of Thr75-DARPP-32 (Brown et al., 2005; Santini et al., 2007), as we observed here in the MPTP mice. These findings suggest that the glutamate/Cdk5/DARPP-32-Thr75 pathway would be important in considering the pathophysiology of PD. Given the novel finding that dopamine signals negatively regulate expression of Cdk5-pTyr15, an active form of Cdk5, our present results contribute to a further understanding the mechanism by which striatal dopamine deficiency causes parkinsonian symptoms.

L-Dopa therapy still remains as the gold standard for treating PD patients; however, it has a potential risk of troublesome side-effects such as adverse fluctuations in motor responses and L-dopa-induced dyskinesias (LIDs) (Calabresi et al., 2010). Maladaptive synaptic plasticity at glutamatergic synapses coupled with dopamine receptors has been suggested in the development of LIDs (Feyder et al., 2011; Murer and Moratalla, 2011). Exploration of new drugs that could exert anti-PD effects without direct activation of dopamine receptors is therefore prudent. We suggest the possibility that imatinib and other c-Abl inhibitors might serve as an alternative and additional therapeutic tool in treating PD symptoms and PD-associated LIDs.

### Conflict of interest statement

The authors declare that the research was conducted in the absence of any commercial or financial relationships that could be construed as a potential conflict of interest.
